# Assessment of health risk associated with persistent organic pollutants in water

**DOI:** 10.1007/s10661-012-2570-8

**Published:** 2012-03-06

**Authors:** Adam Pawełczyk

**Affiliations:** Wrocław University of Technology, ul. Wyspianskiego27, 50-370 Wroclaw, Poland

**Keywords:** Environmental monitoring, Risk assessment, Polychlorinated biphenyls, Chloroorganic pesticides, Water pollution

## Abstract

This paper presents the results of analyses of water samples taken from water intakes supplying one of the districts located near the city of Wrocław. Surprisingly high concentrations of polychlorinated biphenyls and chloroorganic pesticides, classified as persistent organic pollutants (POPs), were detected in the monitored sites. Basing on the analytical and toxicological data, the individual health risks related to carcinogenic effects (excess cancer risk over a lifetime) in humans were assessed, resulting from direct ingestion of community water. Also noncarcinogenic effects resulting from exposure to the examined POPs were determined. The conservative approach to risk assessment, taking into account a safety margin for data incompleteness, was adopted. The carcinogenic risk was found to slightly exceed the unconditionally acceptable risk of 10^−6^ in the case of polychlorinated biphenyls and hexachlorocyclohexane, for all the inhabitant populations. The determined values of noncarcinogenic effects expressed by hazard quotient and hazard index indicate that the water pollutants and their concentrations do not cause an increase in noncarcinogenic incidences in the inhabitants using the monitored water sources.

## Introduction

Polychlorinated biphenyls and chloroorganic organic pesticides used on a massive scale in not so distant past still pose major health hazard to people and animals. Polychlorinated biphenyls are a mixture of congeners differing in their composition. Theoretically, 209 congeners produced by the chlorination of biphenyl are possible (Mills et al. 2007a). These compounds have different trade names, such as: Aroclor in the USA, Phenochlor in France, Clophen in Germany, Kanechlor in Japan, Fenchlor in Italy, Sovol in Russia and Delor in the former Czechoslovakia. Industrial polychlorinated biphenyl preparations called Chlorofen and Tarnol were produced in Poland. Polychlorinated biphenyls are characterized by low reactivity, are fire-retardant and poorly biodegradable (Falandysz et al. 2007). The recommended chemical nomenclature of polychlorinated biphenyl congeners used further in this work is referenced to (Mills et al. 2007). It was mainly owing to these properties that polychlorinated biphenyls (PCBs) were commonly and widely used in industry. Polychlorinated biphenyls were chiefly used as additives to electrical oils, compressor oils, hydraulic oils, paint improvers, impregnating agents, antidust agents, plasticizers for plastics, rubber softeners, and heat-carrying agents. Because of the proven harmful effect of polychlorinated biphenyls on living organisms their production and use were discontinued. Polychlorinated biphenyls, similarly as chloroorganic pesticides, were labeled as toxic organic compounds TOCs. Similar restrictions were imposed on the use of chlorinated pesticides.

Chlorinated pesticides contributed to a reduction in diseases, higher crops, and increased animal production. Hexachlorocyclohexane and p,p′-dichlorodiphenyltrichloroethane (DDT), as well as the persistent chloroorganic compounds from its decomposition, are still present in the environment, despite the ban on their use introduced in most countries in the 1970s. Over time p,p′-dichlorodiphenyltrichloroethane decomposes into p,p′-dichlorodiphenyldichloroethylene (DDE) and p,p′-dichlorodiphenyl dichloroethane (DDD), which have similar physical, chemical and toxicological properties. As they pass through the food chain, the substances accumulate in human and animal organisms, mainly in their fatty tissue and internal organs. Thirty three years after its banning in the USA, p,p′-dichlorodiphenyltrichloroethane is still detectable in about 10% of the population while DDE occurs in almost everyone (Eskenazi 2006).

The use of polychlorinated biphenyls and pesticides has led to the accumulation of these compounds in the environment and their migration, resulting in the contamination of waters, including drinking water intakes. Polychlorinated biphenyls get into the environment through evaporation and leakages and during incineration, industrial sewage discharge and waste disposal. It is estimated that about 80% of the annual production of polychlorinated biphenyls would get into the environment in this way (Nisbet and Sarofim 1972). Atmospheric conditions play a major role in the transport of chloroorganic pesticides. The concentration of the pesticides in water increases when the thaw begins, whereby they are then detected in mountain waters and even in the polar regions. When they get into the atmosphere, polychlorinated biphenyl vapors are absorbed on the suspended dust particles. The average concentration of polychlorinated biphenyls in air in different areas of Sweden amounted to 1–50 ng/m^3^ (PZWL 1985). In environmental hazard conditions, less than 5% of the dose absorbed by the body comes from absorption through the respiratory tract and the skin. The other 95% of the dose is absorbed through the alimentary canal (Koopman-Esseboom et al. 1994).

Analyses of the water drawn for consumption purposes from the intakes in one of the districts near the city of Wrocław showed a surprisingly high level of its contamination with persistent organic pollutants POP such as polychlorinated biphenyls and chloroorganic pesticides (Pawełczyk et al. 2008) in comparison with standards for drinking water (MHC 2000). The cause of the presence of these pollutants in the investigated water intakes has not been explained yet. Probably the polychlorinated biphenyls infiltrate into water mainly in places where industrial and municipal sewage is discharged into rivers.

Solubility of polychlorinated biphenyls is low and decreases with increasing chlorine content. Sewage treatment does not remove dissolved fraction of polychlorinated biphenyls from water, therefore they pass from the sewages to various water reservoirs. Due to hydrophobic properties, polychlorinated biphenyls tend to absorb on suspended solid organic and inorganic particles and only that part of PCBs can be easily separated from water.

Dissolved polychlorinated biphenyls pass also through commonly used raw water treatment systems (Starek 2001). The widespread sand beds are not capable of adsorbing the dissolved organic compounds. For that purpose, activated carbon systems or other techniques would be suitable, which increase costs of the water-treatment processes.

The main objective of the work was to determine the contribution of selected POP contaminants present in the water intakes, to excess adverse health effects, among the local population consuming the water. Actually, the so-called Excess Lifetime Cancer Risk (ELCR) for cancer effects and the so-called hazard quotient for noncancer effects originating from polychlorinated biphenyls and pesticides in water were discussed. The research should determine if any measures have to be taken in order to abate the exposure of the population to these particular contaminants’ groups.

The health risk assessment presented in the paper was conducted according to the procedure recommended by the US Environmental Protection Agency (US EPA) in Risk Assessment Guidance for Superfund (EPA 1989). It includes hazard identification, dose–response assessment, exposure assessment, risk characterization, and uncertainty analysis.

## Materials and methods

Raw water taken from three deep wells located in the vicinity of a county town near Wrocław was analyzed. Also, water samples taken from a small river flowing through the protected area of the district’s water intakes were examined. The chemical analyses showed that congeners: 2,2′,5-trichlorobiphenyl (PCB18), 2,3,3′-trichlorobiphenyl (PCB20), 2,4,4′-trichlorobiphenyl (PCB28), 2,2′,3,5′-tetrachlorobiphenyl (PCB44), 2,2′,5,5′-tetrachlorobiphenyl (PCB52), 2,2′,4,5,5′-pentachlorobiphenyl (PCB101), 2,3′,4,4′,5-pentachlorobiphenyl (PCB118), 2,3,3′,4,4′-pentachlorobiphenyl (PCB105), 2,2′,4,4′,5,5′-hexachlorobiphenyl (PCB153), 2,2′,3,4,4′,5′-hexachlorobiphenyl (PCB138), 2,2′,3,3′,4,4′,5-heptachlorobiphenyl (PCB170), 2,2′,3,4,4′,5,5′-heptachlorobiphenyl (PCB180), and 2,2′,3,3′,4,4′,5,5′-cctachlorobiphenyl (PCB194) were present in the water intakes. Also, chloroorganic pesticides were found in the water.

The material was taken into glass containers every 2 weeks and transported to an analytical laboratory. The adapted analytical procedures according to the Polish Standards (PS 2002) were applied for determination of the examined pollutants in water. After a standard solution was added, double extraction with *n*-hexane was performed. Each sample was purified by adding sulfuric acid, shaking and separating the layers. The extract was dried by filtering it through a layer of sodium sulfate into a round-bottom flask, and concentrated (Pawełczyk et al. 2008).

Then a potassium hydroxide solution was added, the sample was heated and ethyl alcohol was introduced. After reshaking, the *n*-hexane layer was separated. The extracted and purified samples were analyzed using an M504 gas chromatograph equipped with an HP5 capillary column and an ECD electron capture selective towards chloroorganic compounds. To heighten the detectability range, sensibility of the apparatus was adjusted. The chromatograph’s operating parameters are shown in Table 1. Except of DDE, the lower quantification limits both for polychlorinated biphenyls and the pesticides were 1 ng/dm^3^. For p,p′-dichlorodiphenyldichloroethylene the limit was 5 ng/dm^3^.

**Table 1:** Parameters of chromatographic apparatus

Parameter	Value/type
Column length	60 m
Column temperature	
Initial	160°C
Temperature rise	5°C/min
Final	325°C
Initial isotherm	1 min
Final isotherm	15 min
Carrier gas	Nitrogen
Doping gas	Nitrogen

## Results of water analyses

Tables 2 and 3 show the averaged results of analyses for the persistent organic pollutants monitored in the three water intakes and in the river flowing through the water-bearing areas in the period of 5 months. The samples were collected every week; that means the each average value was obtained from about 20 determinations. The average contents of PCBs and chloroorganic pesticides from the three wells were 451.96 and 73.53 ng/dm^3^, respectively.

**Table 2:** Qualitative and quantitative polychlorinated biphenyls composition of water samples from three wells and river (nanogram per cubic decimeter)

Analyte	CAS No	Well I	Well II	Well III	Average I-III	River
PCB18 *2*,*2*′,*5-trichlorobiphenyl*	37680-65-2	2.44	285.87	131.33	139.88	44.31
PCB20 *2*,*3*,*3*′-*trichlorobiphenyl*	38444-84-7	0.78	15.29	0	5.36	672.74
PCB28 *2*,*4*,*4*′-*trichlorobiphenyl*	7012-37-5	128.23	6.27	241.62	125.37	–
PCB44 *2*,*2*′,*3*,*5*′-*tetrachlorobiphenyl*	41464-39-5	167.65	8.56	118.66	98.29	485.27
PCB52 *2*,*2*′,*5*,*5*′-*tetrachlorobiphenyl*	35693-99-3	0	0.14	179.46	59.87	–
PCB101 *2*,*2*′,*4*,*5*,*5*′*-pentachlorobiphenyl*	37680-73-2	0	3.44	8.53	3.99	2.18
PCB118 *2*,*3*′,*4*,*4*′,*5-pentachlorobiphenyl*	31508-00-6	6.12	1.17	0	2.43	–
PCB105 *2*,*3*,*3*′,*4*,*4*′*-pentachlorobiphenyl*	32598-14-4	0.03	7.85	3.21	3.70	1.46
PCB153 (*2*,*2*′,*4*,*4*′,*5*,*5*′-*hexachlorobiphenyl*)	35065-27-1	0	1.67	7.22	2.96	6.74
PCB138 *2*,*2*′,*3*,*4*,*4*′,*5*′-*hexachlorobiphenyl*	35065-28-2	7.62	4.13	4.02	5.26	8.54
PCB170 *2*,*2*′,*3*,*3*′,*4*,*4*′,*5-heptachlorobiphenyl*	35065-30-6	5.43	8.07	0.69	4.73	2.13
PCB180 *2*,*2*′,*3*,*4*,*4*′,*5*,*5*′-*heptachlorobiphenyl*	35065-29-3	0	0.11	0	0.04	–
PCB194 *2*,*2*′,*3*,*3*′,*4*,*4*′,*5*,*5*′-*octachlorobiphenyl*	35694-08-7	0	0	0.24	0.08	7.34
Total		318.30	342.57	694.98	451.96	1,230.71

**Table 3:** Chloroorganic pesticides contents in water samples from three wells and river (nanogram per cubic decimeter)

Analyte	CAS number	Well I	Well II	Well III	Average I–III	River
1,2,3,4,5,6-Hexachlorocyclohexane (gamma-HCH)	58-89-9	3.26	98.17	65.74	55.72	153.86
p,p′-Dichlorodiphenyldichloroethylene (DDE)	72-55-9 (p,p′-DDE)	6.21	4.84	0	3.68	0.02
p,p′-Dichlorodiphenyl dichloroethane (DDD)	72-54-8 (p,p′-DDD)	10.48	2.85	4.65	5.99	7.48
p,p′-Dichlorodiphenyltrichloroethane **(**DDT)	50-29-3	14.39	6.65	3.31	8.12	6.63
Total		34.34	112.51	73.70	73.53	167.99

The concentrations of the chloroorganic compounds vary between the particular intakes. Besides the water from the river, the highest pollutants’ contents occur in intake III. From among polychlorinated biphenyls, the investigated intakes contain most 2,2′,5-trichlorobiphenyl and 2,2′,3,5′-tetrachlorobiphenyl and from among pesticides, they contain most 1,2,3,4,5,6-hexachlorocyclohexane (gamma-hexachlorocyclohexane; gamma-HCH). The average pollutants’ contents calculated for all the intakes for the whole monitoring period amounts to 451.95 and 73.53 ng/dm^3^ for polychlorinated biphenyls and pesticides, respectively (Pawełczyk 2010). Some of the given values are very insignificant and below the quantification limit. However, they were given to realize that certain compounds were detected in water in very low concentrations, but they should not affect the risk level in any meaningful way. No regularity and systematic trends were observed that could be a ground for reasonable conclusions related to the concentrations’ anticipation.

## Hazard identification

Reports on possible adverse health effects caused by polychlorinated biphenyls and chloroorganic pesticides and the products of their decomposition arouse much interest among specialists involved in the assessment of the health risk connected with the presence of these pollutants in the environment. The epidemiological assays showed that the persistent organic pollutants negatively affect living organisms and disturb their hormonal balance and normal biological functioning. As a result, reproductive organs failures, immunological system handicaps, tumor and cancer formation, and defects of major internal organs can occur. In many cases, the consequences of the exposure to the pollutants appear after a long time, sometimes in maturity or in the subsequent generations.

The monitored substances are classified into group B2 (possibly carcinogenic to humans), except for gamma-HCH which has not been classified by EPA. None of the pollutants detected in the water was classified into group A (carcinogenic to humans).

EPA has set an enforceable regulation for polychlorinated biphenyls, called a maximum contaminant level MCL, at 0.0005 mg/dm^3^ (EPA 1992b). The European Council Directive 98/83/EC sets the standards for pesticides at 0.0001 mg/dm^3^, and pesticides total at 0.0005 mg/dm^3^ (EU 1998). The polychlorinated biphenyls are not regulated in the directive.

The current standards for particular pesticide level in drinking water set by the Polish Ministry of Health Care are consistent with the European regulations and amounting to 0.1 μg/dm^3^ (0.0001 mg/dm^3^), and 0.0005 mg/dm^3^ for total pesticides level (MHC 2007). The regulation does not mention the permissible polychlorinated biphenyl level but the previously obligatory document specified 0.5 μg/dm^3^ (0.0005 mg/dm^3^) of polychlorinated biphenyls as a standard for the drinking water (MHC 2000).

## Determination of dose–response relation

The dose–response relation assessment has been limited to oral exposure of the people affected because ingestion was regarded as a crucial exposure pathway for the polychlorinated biphenyls and pesticides present in the water.

Among the general population of the district concerned, children in different ages and a population of women and men (forming a population of adults (*j*)) are distinguished. The structure of the population is shown in Table 4.

**Table 4:** Structure of general population in absolute numbers and in percentage (GUS 2004)

Population (*j*)					
1	2	3	4	5	6
Children 0–6 years old	Children 7–17 years old	Adults (4 + 5)	Men	Women	General population
934	2,046	8,140	4,192	3,948	11,120
8.4%	18.4%	73.2%	37.7%	35.5%	100%

The population uses the municipal system of water supply, drawing water from the three wells examined. The drawn water is treated at the water-treatment station and delivered by drinking water pipes to customers.

As the contaminants concerned are suspected to produce both toxic (noncarcinogenic) and carcinogenic adverse health effects, the dose–health response relation was determined for both types of effects, on the basis of the available toxicity data. Table 5 shows the characteristics of the monitored POPs: RfD, no observable adverse effect level (NOAEL)—for toxic effects and SF_0_, and carcinogen classification, according to (EPA 1986a)—for carcinogenicity. Determination of dose–response relation is conducted separately for carcinogenic and noncarcinogenic effects caused by the POPs absorbed from water.

**Table 5:** Carcinogenic classification, SF_0_ values and toxicity of monitored polychlorinated biphenyls, according to EPA, IRIS (EPA 1986a; OEHHA 2006)

Pollutant	US EPA carcinogens *IARC*	SF_0_ (mg/(kg⋅day))^−1^ *IRIS*	RfD mg/(kg⋅day)	NOAEL mg/(kg⋅day)
∑ PCBs	B2, Probable	2.0^*^	2 ⋅ 10^−5^	0.007 (Aroclor 1016)
1,2,3,4,5,6-Hexachlorocyclohexane (gamma-HCH)		1.10E + 00 (RAIS)	3 ⋅ 10^−4^ (gamma HCH)	0.33
p,p′-Dichlorodiphenyldichloroethylene (DDE)	B2, Probable	3.40E-01	3 ⋅ 10^−3^	
p,p′-Dichlorodiphenyl dichloroethane (DDD)	B2, Probable	2.40E-01	9 ⋅ 10^−3^	
p,p′-Dichlorodiphenyltrichloroethane (DDT)	B2, Probable	3.40E-01	5 ⋅ 10^−4^	0.05

*NOAEL* no observable adverse effect level

### Dose–carcinogenic response relation

Because of the differential ability of different polychlorinated biphenyl mixtures to cause cancer, US EPA developed a range of cancer slope factors based on Aroclors 1016, 1242, 1254, and 1260. These include the range of typical congeners found in various environmental media such as water and fish (OEHHA 2006). In Table 5, the cancer potency of polychlorinated biphenyls mixture is given (IRIS 1997).

For the carcinogenic effects, the health risk was estimated using the following formula:

1 $$ {\text{Risk}} = {\text{CDI}} \cdot {\text{CSF}} $$

where,

- risk—unitless cancer probability of developing cancer,
- CSF—slope factor, expressed in (milligram per kilogram per day)^−1^.
- CDI—chronic daily intake averaged over 70 years mg/(kg⋅day)—ingested with drinking water, by inhalation with air, by ingestion of contaminated fruits and vegetables, by accidental ingestion of contaminated soil or by dermal contact with the contaminated soil, etc. CDI is also called the lifetime average daily dose (LADD).

The linear Eq. (1) is valid only when low risk levels exist, that is below 0.01. For higher exposure levels where higher risk is expected the following expression is used:

2 $$ {\text{Risk}} = 1 - {e^{{ - \left( {{\text{CDI}} \cdot {\text{CSF}}} \right)}}} $$

Cancer risk for multiple substances can be calculated from the following equation:

3 $$ {\text{Ris}}{{\text{k}}_t} = \sum\limits_{{i = 1}}^m {{\text{ris}}{{\text{k}}_i}} $$

where:

- risk_*t*_—the total cancer risk expressed as a unitless probability
- risk_*i*_—the risk estimate for the *i*th substance.

The calculated risk represents the probability of cancer incidence above the natural level in the environment at particular site, caused by the contaminating chemical.

Actually, in the present work, the so called excess cancer risk over a lifetime ELCR, produced by oral intake of polychlorinated biphenyls and chloroorganic pesticides (COPs) with water has been determined instead of the general risk. “Excess” means that additional cancer risk cases in the population are calculated, which should be totaled up with the background risk to get absolute cancer risk. This approach mathematically calculates the probability of developing cancer over a person’s lifetime at a given exposure level. It is expressed by a value representing the number of extra cancer cases expected in a given number of people on exposure to a carcinogen at a stated dose. The ELCR concept was developed by the (EPA 1986a; ENSR 2005). For the present case

4 $$ {\text{Risk}} = 1 - {e^{{ - \left( {{\text{LADD}} \cdot {\text{CS}}{{\text{F}}_o}} \right)}}} $$

where,

- LADD—lifetime average daily dose or intake of a pollutant with drinking water averaged over the lifetime (per milligram per kilogram body weight)
- CSF_0_—an oral cancer slope factor (milligram per kilogram per day)^−1^.

The ELCR value expresses the probability of an extra cancer case, e.g. 1**⋅**10^−6^ means one extra cancer case per one million people.

In the case of exposure to several carcinogenic substances, what is the case in the present study, the total risk is assessed in accordance with the principle of the cumulative effect of carcinogens on the body, by adding the risks calculated for the individual carcinogens:

5 $$ {\text{ELC}}{{\text{R}}_{\text{tot}}} = \sum\limits_{{i = 1}}^m {{\text{ELC}}{{\text{R}}_i}} $$

where:

- ELCR_tot_—the total excess risk of occurrence of carcinogenic effects, caused by all the substances.
- ELCR_*i*_—the excess risk assessed for the *i*th substance.

### Dose–noncarcinogenic response relation

In case of the toxic, noncarcinogenic effects caused by the polychlorinated biphenyls and COPs; not risk, but hazard quotient (HQ) was determined. The HQ has nothing to do with the risk. It is calculated by comparing the average daily doses ADD of a pollutant taken in, with the reference doses RfD. The hazard quotient is expressed by the formula (6) (EPA 1992a):

6 $$ {\text{HQ = ADD/RfD}} $$

The ADD exposure level (intake) expressed in milligram per kilogram per day is calculated differently, depending on the exposure durations. For the lifetime period, it is equivalent to the lifetime average daily dose LADD. For shorter durations, appropriate averaging times, not the lifetime, must be used. Also, reference doses RfDs have to be consistent with the exposure duration which means that the used RfD values should be determined for appropriate exposure periods.

For exposure to multiple noncarcinogenic substances, the aggregate potential for the noncarcinogenic health effects—the hazard index (HI) was calculated from the formula (7).

7 $$ {\text{HI}} = \sum\limits_{{i = 1}}^m {{\text{H}}{{\text{Q}}_i}} $$

where, HQ_*i*_ is a hazard quotient for the *i*th substance. An HI exceeding 1 means that at a given exposure value harmful health effects may arise.

## Exposure assessment

The aim of this step of the health risk assessment was to quantify exposure to the analyzed polychlorinated biphenyls and COPs. It means that the taken in doses were determined in compliance with the assumed resident’s scenario. As the chemicals considered demonstrate both toxic and carcinogenic effects, the quantification was conducted separately for both effects. Since it is mainly the inhabitants using the water supply system who are exposed to polychlorinated biphenyls and COPs found in the water intakes, potential health effects assessment was limited to the resident scenario. The particular populations and the general population are shown in Table 4. The exposure path was the intake of the pollutants through the alimentary tract.

The EPA Guidance for Superfund advises the upper-bound value of 30 years for exposure duration (90th percentile at one residence) when calculating maximum residential exposures MRE. In some cases, lifetime exposure, that is 70 years by convention, may be a more appropriate assumption (EPA 1989). Thus, the exposure durations for all subpopulations considered and for carcinogenic and noncarcinogenic effects are as shown in Table 6.

**Table 6:** Exposure durations and averaging times for carcinogenic and noncarcinogenic effects within the subpopulations, for the resident’s scenario

Population	Children 0–6	Children 7–17	Adults
Carcinogenic effects			
Exposure duration (ED)—years	6	11	30
Averaging time (AT)—days	25,550	25,550	25,550
Noncarcinogenic effects			
Exposure duration (ED)—years	6	11	30
Averaging time (AT)—days	2,190	4,015	10,950

### Exposure assessment for carcinogenic effects

For carcinogenic effects and the resident’s scenario the lifetime average daily dose, LADD can be calculated from relation derived from formula (8) (EPA 1992a; Božek et al. 2009):

8 $$ {\text{LADD = }}\overline {\text{c}} \,{\text{xFIx}}\left( {\text{IRxEFxED}} \right){/}\left( {\text{BWxAT}} \right)\,\left[ {{\text{mg}} \cdot {\text{k}}{{\text{g}}^{{ - 1}}} \cdot {{\text{d}}^{{ - 1}}}} \right] $$

where:

- $\overline c$ represents the average concentration of polychlorinated biphenyls and chloroorganic pesticides in the wells during the monitoring period (nanogram per cubic decimeter).
- Fraction ingested (FI) is an absolute number in a range of 0–1, specifying the contribution of the particular pollution sources to the pollutant intake by people. According to estimations made on the basis of data of the Food Surveys Research Group (Sebastian et al. 2011), about 65% of the consumed water is plain tap water. The remaining 35% is bottled water. Similar proportion was applied in the present work, thus, the FI was assumed 0.65.
- IR represents the intake rate of the specific medium (water). For the particular age groups the daily water intake rates according to (ECETOC 2001) are as follows: 0–6, 0.3 dm^3^/day; 7–17, 1 dm^3^/day; adults, 1.4 dm^3^/day.
- EF is the exposure frequency = 365 days/year.
- ED—the exposure duration. It depends on the age group and for the children from group 0–6 it is 6 years; for the 7–17 group, it is 11 years. For adults, the upper-bound value of 30 years was applied for the lifetime period, as recommended by EPA (1989).
- BW—the average body weight of 15 kg for the 0–6 age group, 46 kg for the 7–17 age group, and 70 kg for adults (average; EPA 1989).
- AT—the averaging time = 70 years **⋅** 365 days/year = 25 550 days for all age groups. The AT was applied according to Superfund guidelines but some authors suggest applying the averaging times equal to exposure durations, when concentrations are approximately constant in considered period of time (Božek et al. 2009). In such case, the calculated risks are significantly higher. The shorter the exposure period, the bigger differences exist between calculated values among the both approaches.

The calculated LADDs are shown in Table 7, as a matrix A of type *m* × *n*, where *m*, *n* $\varepsilon$ $\mathbb{N}$ , whose elements are doses taken in with water through the alimentary tract. In *a*_*ij*_, the matrix elements, index “*i*” represents the type of a pollutant while index “*j*” represents a population distinguished from the general population. The LADD values are valid for the resident scenario for which expose durations and averaging time are given in Table 6.

**Table 7:** Individual lifetime average daily doses (LADD) of polychlorinated biphenyls and COPs, taken in by individuals in different populations calculated from formula (8) [milligram per kilogram per day]

Pollutants	LADD_0–6_	LADD_7–17_	LADD_Adult_
Polychlorinated biphenyls	5.036E-07	1.004E-06	2.518E-06
1,2,3,4,5,6- Hexachlorocyclohexane (gamma-HCH)	6.209E-08	1.237E-07	3.105E-07
p,p′-Dichlorodiphenyldichloroethylene (DDE)	4.104E-09	8.179E-09	2.052E-08
p,p′-Dichlorodiphenyl dichloroethane (DDD)	6.678E-09	1.331E-08	3.339E-08
p,p′-Dichlorodiphenyltrichloroethane (DDT)	9.044E-09	1.802E-08	4.522E-08

### Exposure assessment for noncarcinogenic effects

For calculations of noncarcinogenic chemicals, intakes for resident’s scenario formula similar to (8) was used, but instead of the lifetime average daily doses, average daily doses ADD were determined:

9 $$ {\text{ADD = }}\overline {\text{c}} \,{\text{xFIx}}\left( {\text{IRxEFxED}} \right){/}\left( {\text{BWxAT}} \right)\left[ {{\text{mg}} \cdot {\text{kg}} \cdot {{\text{d}}^{{ - 1}}}} \right] $$

where particular symbols have the same meaning as in preceding formula (8) but some have different value from those used for calculating LADD. In the case of ADD, the exposure duration ED value depends on the age group. For children from group 0–6 it is 6 years and for the 7–17 group it is 11 years. For adults exposure duration is AD = 30 years.

The values of averaging times AT equal the exposure durations and depend on the age group. They are expressed in days. For the 0–6 age group AT_0–6_ = 2 190, AT_7–17_ = 4 015 and for adults AT_A_ = 10 950 days. The calculated average daily doses ADD are shown in Table 8. The ADD values are valid for the resident scenario for which expose durations and averaging times are given in Table 6.

**Table 8:** Individual average daily doses ADD of polychlorinated biphenyls and COPs, taken in by individuals in different populations calculated from formula (9) [milligram per kilogram per day]

Pollutant	ADD_0–6_	ADD_7–17_	ADD_Adult_
PCBs	5.875E-06	6.386E-06	5.875E-06
1,2,3,4,5,6- Hexachlorocyclohexane (gamma-HCH)	7.244E-07	7.874E-07	7.244E-07
p,p′-Dichlorodiphenyldichloroethylene (DDE)	4.788E-08	5.205E-08	4.788E-08
p,p′-Dichlorodiphenyl dichloroethane (DDD)	7.791E-08	8.469E-08	7.791E-08
p,p′-Dichlorodiphenyltrichloroethane (DDT)	1.055E-07	1.147E-07	1.055E-07

## Risk characterization

Quantification of the impact of considered water pollutants on the resident’s health has been carried out on the basis of the absorbed doses, separately for carcinogenic and toxic effects. The calculated health risk levels related to carcinogenic effects and comparison of the average daily doses with reference doses in case of toxic effects enabled to determine the health thread level the population is facing in the area operated by the local water system supply.

### Cancer risk characterization

Table 9 shows excess lifetime cancer risks ELCR separately for the individual pollutants and populations and the total risk for all the pollutants, occurring in the particular populations, calculated from formulas 4 and 5. The total risk represents the total probability of cancer incidence above the level natural in the environment devoid of the investigated pollutants.

**Table 9:** ELCR_ind-i_ values resulting from individual pollutants, calculated from Eq. (4) for particular populations, and ELCR_tot-*j*_ values resulting from cumulative effect of all pollutants, calculated from equation (5) for particular populations

Pollutant	ELCR_0–6_	ELCR _7–17_	ELCR _Adult_
polychlorinated biphenyls	1.007E-06	2.007E-06	5.035E-06
1,2,3,4,5,6- Hexachlorocyclohexane (gamma-HCH)	1.242E-07	2.474E-07	6.209E-07
p,p′-Dichlorodiphenyldichloroethylene (DDE)	8.208E-09	1.636E-08	4.104E-08
p,p′-Dichlorodiphenyl dichloroethane (DDD)	1.336E-08	2.661E-08	6.678E-08
p,p′-Dichlorodiphenyltrichloroethane (DDT)	1.809E-08	3.604E-08	9.043E-08
ELCR_tot-*j*_			
∑ ELCR	1.171E-06	2.333E-06	5.855E-06

A comparison of the values shown in Table 9 with the unconditionally acceptable ones shows that the highest carcinogenic risk is connected with the presence of polychlorinated biphenyls and gamma-HCH in water. In all the populations, the total risk exceeds, although slightly, the level of acceptable risk. In relation to the unconditionally acceptable risk level, ELCR_tot-*j*_ slightly exceeds safety standards in 0–6–children population, 2.3 times in 7–17–children population, and 5.8 times in adults’ population.

From the calculated individual risks, an absolute number of extra cancer cases ECC_pop-*j*_ can be derived, expected in the considered *j*th population, having size *N*_*j*_ (Biesiada 2000).

10 $$ {\text{EC}}{{\text{C}}_{{{\text{pop - j}}}}} = {\text{ELC}}{{\text{R}}_{{{\text{ind - j}}}}} \times {N_j} $$

where,

- ECC_pop-*j*_—extra cancer cases expressed as the total number cancer cases in population “*j*” (children 0–6, children 7–17, women, men, adults, general population, etc.), caused by a pollutant
- ELCR_ind-*j*_—an individual excess cancer risk caused by a pollutant
- *N*_*j*_—the number of people in the “*j*” population

Each ECC_pop-*j*_ value is calculated separately for each of the pollutants. The conversion of an individual risk into extra cancer cases in a population makes sense only for appropriately large populations (in accordance with the law of larger numbers).

Table 10 shows ECC for all the four populations (formula 10): children 0–6, children 7–17, and adults, as an absolute number of extra cancer cases in a given population, due to the presence of a given pollutant. Also the total number of extra cancer cases ∑ECC_pop_ caused by all the substances is shown. The expected absolute number of extra cancer cases in the whole studied population ECC_gen–pop_, due to the contamination of water with all the pollutants, amounts to about 0.053.

**Table 10:** ECC_pop-*j*_ values equivalent to absolute number of excess cancer cases in population “*j*”, due to individual pollutant (*i*), cumulative ECC values as sum of ECC_pop-*j*_ within population ∑ ECC_pop_ and ECC_gen–pop_ equivalent to absolute number of excess cancer cases in whole population

Pollutant	Population		
	Children 0–6	Children 7–17	Adults
	ECC_pop-1_	ECC _pop-2_	ECC _pop-3_
Polychlorinated biphenyls	9.406E-04	4.106E-03	4.099E-02
1,2,3,4,5,6- Hexachlorocyclohexane (gamma-HCH)	1.160E-04	5.063E-04	5.054E-03
p,p′-Dichlorodiphenyldichloroethylene (DDE)	7.666E-06	3.346E-05	3.341E-04
p,p′-Dichlorodiphenyl dichloroethane (DDD)	1.247E-05	5.445E-05	5.436E-04
p,p′-Dichlorodiphenyltrichloroethane (DDT)	1.689E-05	7.374E-05	7.361E-04
∑ ECC_pop_	1.094E-03	4.774E-03	4.766E-02
ECC_gen–pop_ = 5.34E-02			

The total risks ELCR_tot-*j*_ are compared with the acceptable risk. The acceptable risk level is usually assumed in a range of 1⋅10^−6^ to 1⋅10^−4^ (EPA 1991). The EPA assumes the unconditionally acceptable risk level below 10^−6^ (1:1,000,000; EPA 1986b, 2005). A risk of 10^−3^ absolutely requires protective measures. In general, the US EPA considers excess cancer risks that are below 1 chance in 1,000,000 (1⋅10^−6^ or 1E-06) to be so small as to be negligible, and risks above 1E-04 to be sufficiently large that some sort of remediation is desirable. A comparison of the values with the unconditionally acceptable ones (i.e. 1E-06) shows that the highest carcinogenic risk occurs in the resident scenario for adult group. PCBs contribute most to the excess cancer risk. It exceeds the value 1⋅10^−6^ in all subpopulations but this cannot be a reason for any major concern of residents. The total risks ELCR_tot-*j*_ in the particular subpopulations produced by all the contaminants together are slightly higher than those coming from polychlorinated biphenyls only. This means that the COPs contribution to the total excess cancer risk is insignificant.

### Characterization of noncancerous health effects chances

Table 11 shows a matrix of hazard quotients HQ_*ij*_ due to the contamination of water with polychlorinated biphenyls and COPs, calculated for the particular subpopulations. Also a hazard index for the sum of all the pollutants is given in the table. The values were calculated from formulas 6 and 7 basing on the average daily doses ADD (Table 8) and reference doses RfD (Table 5).

**Table 11:** Hazard quotients HQ_*ij*_ and a hazard index HI_*j*_ for the sum of all the pollutants in water

Pollutant	Population		
	Children 0–6	Children 7–17	Adults
	HQ_*ij*_		
Polychlorinated biphenyls	2.938E-01	3.193E-01	2.938E-01
1,2,3,4,5,6- Hexachlorocyclohexane (gamma-HCH)	2.415E-03	2.625E-03	2.415E-03
p,p′-Dichlorodiphenyldichloroethylene (DDE)	1.596E-05	1.735E-05	1.596E-05
p,p′-Dichlorodiphenyl dichloroethane (DDD)	8.657E-06	9.410E-06	8.657E-06
p,p′-Dichlorodiphenyltrichloroethane (DDT)	2.110E-04	2.294E-04	2.110E-04
PCBs + COPs	HI_*j*_		
	2.964E-01	3.222E-01	2.964E-01

According to Table 11, both the HQ and the HI connected with the water pollutants are below unity. Thus, it can be concluded that the pollutants do not cause harmful noncarcinogenic health effects in the population.

## Uncertainty analysis

When assessing the health risk to people, resulting from the presence of toxic and carcinogenic pollutants in the environment, one should expect certain simplifications and ambiguities, due to which the obtained results cannot be regarded as definite and absolute. The ambiguities and the simplifications are due to the imperfection of the chemical analyses, the simplifying assumptions and the incompleteness of the toxicological data on the substances. The procedure presented here is based on the conservative approach to risk assessment, which tends rather to overestimate than underestimate risk. The main factors contributing to assessment uncertainty are:

- The lack of routine and systematic data from the monitoring of water intakes, which would cover polychlorinated biphenyls and pesticides analyses. It is not known whether the concentrations of the pollutants in the water intakes will remain at a constant level over the whole lifetime of the individuals belonging to a given population.
- The average pollutant concentrations for the three water intakes supplying the inhabitants with water were used in the calculations; however, there are quite significant differences between the water intakes.
- Because of the incomplete data for risk calculation, toxicological data not for pure polychlorinated biphenyls, but for a mixture of congeners were used.
- There is no information on the number of persons in the investigated population, who use other water sources, such as local intakes and water supply systems operated by other drinking water producing plants. For that reason, an estimation was applied assuming that the fraction of the plain tap water ingested was 65% of total water consumption. The calculations were limited only to the ingested water without taking into account dermal contact of human body during washing and during taking a bath.
- Because of the lack of data on the absorption coefficients of the considered carcinogenic substances, the coefficients were assumed to be equal to 1 for all the compounds.

## Conclusions

The investigations on the presence of persistent organic pollutants POP in the environment showed quite high concentrations of polychlorinated biphenyls and chloroorganic pesticides in the drinking water intakes supplying one of the districts near the city of Wrocław. The origin of the pollutants is unknown, but one can suppose that despite the fact that their use had been banned several decades ago they still infiltrate from the industrial and agricultural areas where they had accumulated, into the underground waters.

Observation of the pollutants’ levels in the wells, made during the five sampling months did not show any significant regularity or systematic fluctuations, so it was not possible to draw sensible conclusions about the seasonal concentration trends. Deduction on possible trends could be possible providing that systematic long term analyses are carried out.

Although the concentrations of the pollutants do not exceed the values allowable for drinking water, the carcinogenic risk assessment carried out for the district’s different populations and its general population shows an excess risk of cancer ELCR, which in some cases exceeds the unconditionally acceptable level of 10^−6^. The greatest hazard is connected with the presence of polychlorinated biphenyls in the environment, whereas the concentrations of gamma-HCH, DDT, DDD, and DDE do not pose a risk which could be a cause for concern.

As regards the risk of toxic effects, the calculated hazard quotient HQ and hazard index HI values do not give cause for concern. Under the current toxicity standards, the investigated water intakes are completely safe as regards noncarcinogenic health effects.

It should be stressed, that in case of carcinogenic effects in all subpopulations, the calculated values are valid for the EPA approach that is for averaging times equal to lifetime. When other authors approach is applied, e.g., (Božek et al. 2009), assuming the averaging time equal to exposure time, much higher ELCR values are obtained, particularly in 0–6 years children population. Also the expected absolute number of extra cancer cases in the whole studied population ECC_gen–pop_ is higher than that calculated according to (EPA 1989). It amounts to about 0.091, while the ECC_gen–pop_ calculated in compliance with EPA rules is 0.053.
